# Online glucose-lactate monitoring and control in cell culture and microbial fermentation bioprocesses

**DOI:** 10.1186/1753-6561-7-S6-P18

**Published:** 2013-12-04

**Authors:** Henry Weichert, Mario Becker

**Affiliations:** 1 Sartorius Stedim Biotech GmbH, August-Spindler-Strasse 11, 37079 Goettingen, Germany

## Introduction

Conventional biopharmaceutical manufacturing is characterized by validated process steps and extensive lab testing procedures. The FDA PAT-Guidance recommends the use of potential for improving development, manufacturing, and quality assurance through innovation in product and process development, process analysis and process control.

Measurement of glucose, as a major nutrient during cell cultivation and microbial fermentation, has a key role for controlling the status of the cultivation process. Together with the amount of lactate and additional process parameters, like pH and DO, it gives the possibility to calculate specific consumption rates of nutrients. The user gets information about the status of the culture and of the cells.

## BioPAT^®^Trace: online Glc/Lac analyser

**Figure 1:**
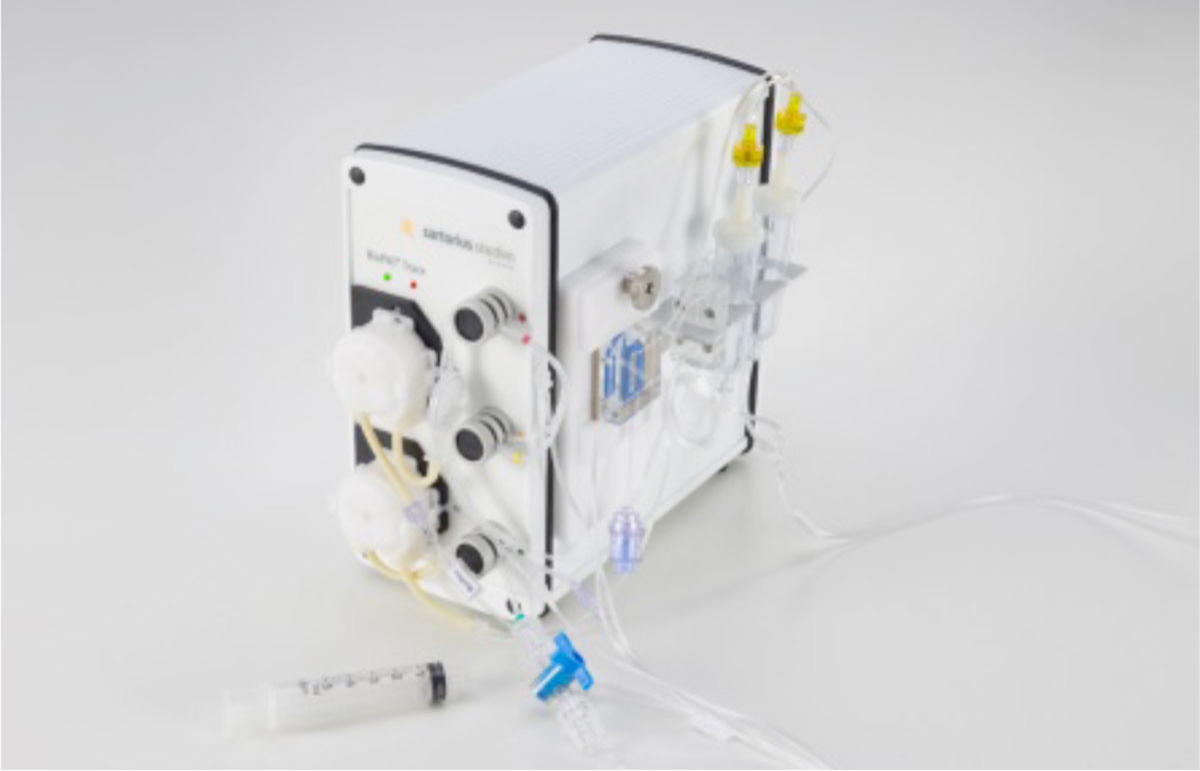
**BioPAT^®^Trace equipped with the single use Tube set**.

BioPAT^®^Trace (Figure 1) is a dual-channel analyser for the simultaneously measurement of glucose and lactate which is based on an enzymatic detection of the two analytics.

Special attention has been paid to the ease of use and hygienic issues related to cGMP environments. The system follows the plug & plays principle, can be fully integrated into all facility environment scenarios and is compliant with all relevant regulatory guidelines.

### Wide measuring range

The linear measuring range of the BioPAT ^®^Trace extends from 0.01 to 40 g/l glucose and from 0.05 to 5 g/l lactate. The deviation from the average measurement value is less than 3% for a measurement of 5 g/l glucose and 2.5 g/l lactate.

### Fast measurement frequency

The measurement frequency is up to 60 analyses per hour depending on the conditions. The service life of the sensor system ensures 30 days or 5000 analyses depending on the application. The ambient temperature of the BioPAT ^®^Trace can lie between 5 and 35°C due to internal temperature correction. The ambient humidity should not exceed 90%.

### Flexible system integration

The BioPAT ^®^Trace has a number of outputs making integration into data recording systems very flexible. Along with a standard analog output for signal ranges from 0 to 20 mA, 0 to 10 V or 4 to 20 mA, the BioPAT ^®^Trace also has a USB interface, an Ethernet connection as well as a serial output for data recording.

### Connection to different fermenter scales by filtration or dialysis probes

The on-line analysing system BioPAT^®^Trace covers the different demands of long-term cell culture cultivations and fast microbial processes in different scales such as small volume cultivations and FDA-validated large scale productions. The sterile sampling systems based on filtration, dialysis or ContiTRACE disposable probes provide the perfect solution for reliable on-line sampling in bioreactors and bio disposables applied in industrial and laboratory facilities.

The simplest method is to directly measure a filtered sample of medium. However, because reactor medium is used, the range of applications is limited to processes for which there's a sufficient reactor volume or which allow continuous-feed. Dialysis sampling is an option when processes are involved for which reactor volume does not allow enough sample material.

This method only removes low molecular substances from the reactor medium, without reducing the volume of fluid.

### Automated control loop for glucose feed

Integrated in an automation platform enabled with a 2 point glucose controller, e.g. as part of an S88 recipe module of the BioPAT^®^MFCS SCADA system, it is possible to realize a fully automated control loop for any kind of cultivation process.

## Conclusions

• Real Online system

Fast & automated measurement

SU tube sets and sensors

• Direct culture control (24/7)

Process knowhow

Replace offline methods

Real-time process monitoring

Automated sampling

• Setup of control loops and event based actions

defined by using the S88 module

• Different connections to automation systems possible

• Automated feed control

• Real-time Glucose and Lactate values

